# TRANSITIONS IN MEDICATION MANAGEMENT AMONG PATIENTS WITH DEMENTIA: PATIENT-CAREGIVER PERSPECTIVES

**DOI:** 10.1093/geroni/igad104.3671

**Published:** 2023-12-21

**Authors:** Rachel O’Conor, Andrea Russell, Dianne Oladejo, Sarah Filec, Darby Morhardt, Emily Rogalski, Lee A Lindquist, Michael Wolf

**Affiliations:** Northwestern University, Chicago, Illinois, United States; Northwestern University, Chicago, Illinois, United States; Northwestern University, Chicago, Illinois, United States; Feinberg School of Medicine, Northwestern University, Chicago, Illinois, United States; Northwestern University Feinberg School of Medicine, Chicago, Illinois, United States; Northwestern University, Chicago, Illinois, United States; Northwestern University, Chicago, Illinois, United States; Northwestern University, Chicago, Illinois, United States

## Abstract

Older adults with Alzheimer’s disease and related dementias (ADRD) have higher rates of multimorbidity and polypharmacy. Medication adherence is critical to maintain health but is difficult with complex regimens. Little is known about how older adults with varying levels of cognitive impairment self-manage medications, and how responsibilities transition to caregivers. We conducted qualitative interviews with patient-caregiver dyads exploring how they managed multidrug regimens. Patients were eligible if they had 1) mild cognitive impairment (MCI), mild ADRD or moderate ADRD, 2) ≥3 chronic conditions, 3) ≥5 medicines and 4) a caregiver. Three coders applied a priori codes to transcripts and analyses followed the framework method. We interviewed 60 participants (Patients: mean age 78 years, 63% female, 50% white, 33% Black; Caregivers: mean age 65 years, 73% female, 60% spouse, 33% child). Patients took 9 medicines on average; cognitively, 36% had MCI, 40% mild ADRD and 23% moderate ADRD. Interviews revealed a tension between preservation of patient independence with early caregiver involvement. Patients and caregivers reported a strong preference towards patient autonomy in medication management, and patients with MCI and mild ADRD managed their medications independently. Among patients with moderate ADRD, caregivers assumed all medication responsibilities except when living separately. In those scenarios, caregivers set up organizers and made reminder calls, but did not observe medication intake. Transitions in medication management followed patient-driven medication errors that resulted in adverse events. These findings have important implications; clinicians should initiate conversations with caregivers and patients about early medication assistance and work to simplify regimens.

